# Sexual Selection as a Driver of Shared Neural and Reproductive Mechanisms

**DOI:** 10.3390/ijms27156793

**Published:** 2026-07-29

**Authors:** Hasan Khatib

**Affiliations:** Department of Animal and Dairy Sciences, The University of Wisconsin, Madison, WI 53706, USA; hkhatib@wisc.edu

**Keywords:** evolution, mate choice, cognition, reproduction

## Abstract

Sexual selection theory provides a unifying framework for understanding how cognitive traits, reproductive strategies, and molecular mechanisms coevolve to maximize fitness. Across species, evidence shows that cognitive performance predicts reproductive success through both male–male competition and female mate choice, with individuals displaying superior problem-solving, social dominance, or courtship skills achieving greater mating success. Sexual selection also favors alternative strategies, including sexual mimicry and socially induced sex change, which allow individuals to navigate competitive mating environments and optimize reproductive output. At a mechanistic level, the close evolutionary coupling of cognition and reproduction is reflected in shared cellular, molecular, and genetic architectures between the brain and reproductive tissues, particularly sperm. Conserved genes and pathways involved in signaling, metabolism, epigenetic regulation, and stress responses link neural function to gamete performance across species. We hypothesized that natural selection on cognitive or reproductive traits drives coordinated evolution of shared genetic and molecular pathways between the brain and reproductive tissues, resulting in conserved mechanisms that support both behavioral and fertilization success. To test this hypothesis, we investigated the extent to which the brain and reproductive tissues share conserved genes and molecular pathways, and whether these shared mechanisms provide evidence for coordinated evolution between cognitive and reproductive functions.

## 1. Introduction to Sexual Selection Theory

Sexual selection, first proposed by Charles Darwin, describes the evolution of traits that enhance reproductive success through mate choice or competition for mates [1,2,3]. In polygynous mating systems, mate choice is typically expressed by females, whereas competition for mates often occurs among males [2]. Female choice can provide both direct benefits, such as access to resources (e.g., high-quality territories, nests, or food), parental care, or protection, and indirect benefits, including enhanced genetic quality of offspring [1,4]. Despite its role in increasing individual-level reproductive success in changing environments, sexual selection can impose substantial costs on both males and females. These costs include elevated predation risk associated with conspicuous traits (e.g., bright coloration, loud vocalizations, or large ornaments), increased injury or mortality resulting from male–male competition (e.g., antler fights in deer or combat among elephant seals), and ecological constraints that limit female choosiness, potentially leading to mating with suboptimal partners and reduced offspring quality [1]. This review examines whether the brain and reproductive tissues share conserved genetic, molecular, and regulatory mechanisms across species, and whether the available evidence supports the hypothesis that cognitive and reproductive functions have undergone coordinated evolution under sexual selection. We refer to cognitive performance as the effectiveness with which an individual performs mental processes such as learning, memory, attention, problem-solving, and decision-making. Reproductive success is the ability of an individual to produce viable offspring that survive and reproduce, thereby passing its genes to future generations.

To test our hypothesis, we conducted a targeted review of the scientific literature to identify studies reporting shared gene expression patterns, signaling pathways, molecular mechanisms, and cellular functions among the brain, sperm, testes, and ovaries. Rather than attempting an exhaustive review of the extensive literature on cognition and reproduction, we selected representative studies that provided direct evidence of molecular and functional overlap between neural and reproductive tissues. To assess the evolutionary conservation of these relationships, we included studies from several mammalian species, including humans, mice, sheep, cattle, and swine. Additionally, we draw on findings from our recent studies [5,6], which demonstrated that many genes expressed in sperm also play important roles in neural function. We further provide evidence that numerous genetic and regulatory features shared between germ cells and the brain are evolutionarily conserved across multiple mammalian species [7], offering additional support for our hypothesis.

## 2. Literature Search and Selection Strategy

This review was conducted as a narrative synthesis to evaluate whether evidence from behavioral ecology, evolutionary biology, genetics, genomics, reproductive biology, and neuroscience supports the hypothesis that cognitive and reproductive functions have evolved through shared molecular mechanisms under sexual selection. A targeted literature search was performed using PubMed, Web of Science, and Google Scholar to identify studies published through April 2026. Search terms included combinations of the keywords: sexual selection, cognition, cognitive performance, mate choice, male–male competition, reproductive success, sexual mimicry, sex change, brain, testis, sperm, ovary, gene expression, transcriptomics, proteomics, epigenetics, neurogenesis, fertility, and evolutionary conservation. Additional relevant publications were identified through citation tracking of key articles and reviews.

Studies were included if they provided evidence linking cognitive traits, sexual selection, reproductive performance, neural function, or shared molecular mechanisms between neural and reproductive tissues. Particular emphasis was placed on studies reporting common genes, signaling pathways, neurotransmitter systems, epigenetic mechanisms, or conserved molecular functions in the brain, sperm, testes, or ovaries. To evaluate evolutionary conservation, studies from multiple species, including humans, mice, rats, sheep, cattle, swine, birds, reptiles, fish, and invertebrates, were considered when relevant to the central hypothesis. These species were chosen because they are widely used in biomedical and agricultural research and collectively provide a broad comparative framework for evaluating conserved mechanisms linking cognition and reproduction.

Because the objective of this review was to synthesize conceptual and mechanistic evidence rather than estimate effect sizes or conduct a quantitative meta-analysis, we did not apply a formal systematic review framework. Instead, representative studies were selected based on their relevance, methodological rigor, and contribution to understanding the potential evolutionary integration of cognitive and reproductive functions. Findings from our recent transcriptomic, proteomic, and epigenetic studies investigating shared molecular features of neural and reproductive tissues [5,6,7,8] were also incorporated to provide further evidence for the conservation of genes and regulatory pathways that link cognition and reproduction across mammalian species.

## 3. Is There Evidence That Cognitive Performance Predicts Reproductive Success?

One of the first studies to address this question examined satin bowerbirds, *Ptilonorhynchus violaceus*, and assessed the relationship between male problem-solving ability and mating success [9]. Male bowerbirds attract females through a combination of behavior, cognition, and decoration: they build stick bowers, decorate them with blue objects, perform elaborate dances and vocalizations, and engage in problem-solving and deceptive behaviors, such as stealing rival decorations or mimicking females to distract competitors. Keagy and colleagues tested male problem-solving by presenting glued barriers over red objects, which males are highly motivated to remove from their bower [9]. The speed with which a male removed the barrier served as a measure of cognitive performance. The study found a positive correlation between problem-solving ability and mating success, measured as the number and rank of copulations across two successive years. Based on this relationship, the authors suggested that enhanced cognitive performance in male bowerbirds may be a target of sexual selection. Although the study reported a positive association between male problem-solving ability and mating success, it was correlational, relied on a limited number of cognitive tasks, and measured mating success rather than direct reproductive success. Cognitive ability’s influence on reproductive performance has been reported in wild great tits [10,11,12], budgerigars [13], and Australian magpies [14]. Although most studies have focused on male cognitive performance, Ashton et al. reported a positive association between general cognitive ability and reproductive success in female magpies, including the average number of fledglings per year, the number of fledglings surviving to independence, and the number of hatched clutches per year [14]. Arden et al. reported a positive correlation between intelligence and semen quality characteristics, including sperm concentration, count, and motility, in male humans [15]. Although novel, the associations reported by Arden and colleagues were relatively modest, with correlation coefficients ranging from 0.14 to 0.19. Furthermore, as acknowledged by the authors, semen quality was assessed from a single ejaculate, which may not reliably reflect an individual’s overall reproductive capacity because semen characteristics can vary substantially over time. Future studies should therefore incorporate repeated semen collections and use averaged measures of semen quality to provide more robust estimates of male reproductive performance.

## 4. Sexual Selection Through Male–Male Competition

Male red deer use their antlers to assess rivals, defend territories, and compete for access to females, thereby influencing harem (a group of female animals sharing a single mate) size and overall reproductive success. Malo and colleagues investigated whether the elaborateness of red deer antlers evolved through sexual selection by examining associations between antler size and reproductive traits [16]. In a wild population of 198 Iberian red deer, the authors tested correlations between antler size and both testis size (a proxy for sperm production) and sperm velocity (an indicator of sperm quality). They reported significant positive relationships between antler size, sperm velocity, and testis size, traits directly linked to fertilization success. These findings suggest that antler exaggeration may function as a reliable signal of male reproductive quality to females. In addition, antler size may convey information to rival males regarding competitive ability, not only at the behavioral level (fighting capacity) but also at the physiological level, reflecting investment in sperm competition [16].

In Soay sheep, Preston et al. demonstrated that sexually selected traits, including horn length, body size, and overall condition, each independently increase a male’s ability to monopolize receptive females, highlighting their role in male–male competition [17]. They further showed that larger testes, another trait under sexual selection, are independently associated with higher copulation rates and increased siring success. Extending these findings to the molecular level, Johnston et al. identified a specific allele of the *relaxin-like receptor 2* gene that is associated with both increased horn size and greater reproductive success, providing genetic evidence that sexually selected traits can directly translate into fitness advantages [18].

Harcourt et al. examined male–male competition by linking mating system variation to testicular size in chimpanzees, compared with gorillas and orangutans [19]. Unlike gorillas and orangutans, where dominant males largely monopolize access to estrous females, chimpanzees exhibit a multi-male mating system in which several males frequently copulate with the same female. Under these conditions, male reproductive success depends on postcopulatory competition, favoring males that produce and transfer larger sperm loads. Consequently, chimpanzees possess testes that are substantially larger relative to body size than those of gorillas or orangutans, consistent with intense sperm competition. This study made a seminal contribution to sexual selection theory by demonstrating that male–male competition in a polyandrous mating system, such as that of chimpanzees, extends beyond precopulatory interactions to include postcopulatory processes. More broadly, it showed that mating system structure shapes the evolution of sexually selected traits, shifting selection from external weapons and dominance to physiological investment in sperm production.

The examples of red deer, Soay sheep, and chimpanzees demonstrate that reproductive success depends on an individual’s ability to assess rivals, compete for mates, and allocate resources to traits that enhance fertilization success. Understanding these examples is essential for linking sexual selection to cognition because successful competition for mates often requires information processing, social assessment, decision-making, and behavioral flexibility. Thus, selection acting on competitive reproductive strategies may favor cognitive mechanisms that enhance an individual’s ability to navigate social and reproductive challenges, creating an evolutionary connection between cognitive performance and reproductive success.

These studies provide strong comparative and correlational evidence linking male–male competition with the evolution of sexually selected traits, but several important limitations should be considered. A key limitation is that most findings are correlational, making it difficult to infer causality. For example, in red deer and Soay sheep, associations between antler or horn size and reproductive traits (such as testis size or sperm quality) do not demonstrate that these traits directly cause higher reproductive success; instead, they may be jointly influenced by underlying factors such as overall condition, developmental quality, or environmental access to resources. A second limitation is that many studies rely on proxy measures of reproductive fitness, such as testis size, sperm velocity, or copulation rate, rather than direct measures of lifetime reproductive success or genetic paternity across multiple breeding seasons. This can overestimate or underestimate true fitness consequences of sexually selected traits. Future research could address these limitations by integrating experimental and longitudinal approaches. Long-term tracking of individuals across multiple breeding seasons, combined with full paternity assignments using genomic tools, would provide more accurate estimates of lifetime reproductive success.

## 5. Female Mate Choice for Cognitive Traits

Sexual selection theory posits that mate choice is shaped in part by selection on cognitive traits and represents one of the most critical decision-making processes for maximizing reproductive success, including the transmission of “good” genes and the acquisition of parental care [4]. Consistent with this view, substantial empirical evidence in humans indicates that females often prefer males that perform well in problem-solving tasks and creativity [20].

Mate choice is pervasive across the animal kingdom, occurring in invertebrates, fish, amphibians, reptiles, birds, and mammals, including humans. It is expressed through visual, acoustic, chemical, and behavioral cues, underscoring its ubiquity and central role in sexual selection, cognitive evolution, and the generation of biodiversity across species. In humans, Prokosch et al. examined whether male intelligence and creativity influence female mate choice [20]. General intelligence was assessed in a cohort of undergraduate men using standardized tests of verbal ability, including the recall of definitions of difficult words, while creativity was evaluated through a series of behavioral tasks designed to elicit insightful and original responses. Videotaped performances of 15 male participants were then rated by 204 undergraduate women for mate preference. The authors found that both intelligence and creativity significantly influenced women’s preferences in both short-term and long-term mating contexts [20]. However, the evidence for mate choice in humans is scarce. It is challenging to study experimentally due to ethical, methodological, social, and evolutionary constraints that are largely absent from, or easier to control in, non-human animals.

In birds, courtship traits such as song, dance, and coloration are common targets of sexual selection. Manakins provide a striking example, as females evaluate males based on the complexity, coordination, and precision of their elaborate courtship dances. Males display cooperative behavior within age-structured dominance hierarchies, yet mating opportunities are largely restricted to alpha males, allowing females to choose among high-performing dominant males across neighboring territories. Porzio & Mota showed that coloration, dance, and song have largely evolved independently in manakins, suggesting that these traits serve distinct signaling functions during courtship [21]. Their findings indicate that each modality may convey different information to females and has been shaped by partially independent natural and sexual selection pressures over evolutionary time. Peafowl (*Pavo cristatus*) represents another classic example of mate choice in birds. Peahens choose mates based on the size, symmetry, and number of eyespots on the males’ elaborate tail trains, classic targets of sexual selection [22]. Petrie et al. showed that peacocks with larger, more symmetrical trains had higher mating success, indicating that these traits serve as reliable signals of genetic quality [22]. Thus, female mate choice in these cross-species examples likely involves a decision-making process aimed at maximizing female reproductive success. Indeed, female choice is a key driver in the evolution of exaggerated male ornaments, illustrating how sexual selection is correlated with both physical traits and reproductive outcomes.

## 6. Sexual Mimicry

Sexual mimicry is a widespread strategy in which individuals mimic the appearance, behavior, or signals of the opposite sex or of non-mating individuals to gain reproductive advantages. This form of deception has been documented across diverse species, including spotted hyenas, garter snakes, damselflies, lizards, butterflies, and birds. It encompasses mechanisms such as morphological resemblance, behavioral imitation, and chemical signaling. By exploiting perceptual or social biases within the same species, sexual mimicry can reduce aggression, circumvent dominance hierarchies, or increase access to mates, highlighting it as an alternative means by which sexual selection could possibly shape mating strategies [23,24,25].

Cuttlefish provide a compelling example of sexual mimicry as a product of sexual selection operating under intense male–male competition. During mating interactions, males guard females and engage in aggressive displays to exclude rivals, creating strong selection for alternative reproductive tactics. In a qualitative study of cuttlefish mating behavior, Norman et al. showed that smaller males can bypass dominant competitors by mimicking females, using rapid skin pattern changes, arm tucking, and posture shifts to adopt female-like appearances and behaviors, thereby gaining access to guarded females [24]. This “sneaker” strategy enables “inferior” males to achieve fertilization despite competitive exclusion. Even more strikingly, some males simultaneously display different patterns on opposite sides of their bodies, directing male courtship signals toward females while presenting female patterns to rival males, effectively preventing interference [24]. These deceptive behaviors illustrate how sexual selection can favor sophisticated signaling and cognitive strategies, highlighting the role of social and sexual interactions in shaping the evolution of cuttlefish cognition.

Another example is the marsh harrier, which represents a rare avian case of permanent sexual mimicry shaped by sexual selection. In this species, a substantial proportion of adult males retain a female-like plumage throughout life, a strategy thought to reduce intrasexual aggression and the costs of male–male competition. Using simulated territorial intrusions, Sternalski et al. showed that typical males directed aggression primarily toward typical male decoys, while female-like male decoys were treated similarly to female decoys and largely avoided attack [25]. Female-like males also behaved like females in territorial defense, directing aggression toward females rather than males. These findings suggest that permanent female mimicry functions as an alternative mating strategy that minimizes costly competition, illustrating how sexual selection can favor deceptive phenotypes to enhance access to reproductive resources.

Sexual mimicry also occurs in other species, such as reptiles and mammals. In red sided garter snakes (*Thamnophis sirtalis parietalis*), sexual mimicry occurs through physiological feminization, whereby some males release female-specific pheromones that attract rival males. These female-mimicking males gain a reproductive advantage by reducing male–male competition and achieving higher mating success, illustrating sexual selection favoring chemical deception as an alternative mating strategy [23].

Spotted hyenas live in clans structured by linear dominance hierarchies in which adult females consistently dominate adult males. In the *Crocuta crocuta* species, females exhibit a striking form of sexual mimicry through highly masculinized external genitalia, including an elongated pseudo-penis. Female genital masculinization was once thought to result from high androgen levels, but studies have shown that it can occur independently of these hormones [26]. Consequently, researchers proposed that the close resemblance between male and female genitalia may represent an adaptive form of sexual mimicry that reduces aggression toward females, particularly during infancy. Although direct evidence remains limited, anatomical similarity between the sexes and the lack of support for the hormone byproduct hypothesis provide support for this idea [26]. From a sexual selection perspective, the pseudo-penis in the *Crocuta crocuta* species constrains copulation by requiring male cooperation, thereby enhancing female control over mating and influencing both social organization and reproductive dynamics.

Together, these examples demonstrate that reproductive success can depend on the ability to process social information, generate deceptive signals, and modify behavior in response to competitors and potential mates. The effectiveness of these strategies requires integrating neural mechanisms that govern cognition, communication, and social behavior with reproductive traits such as genital morphology, pheromone production, and mating tactics. Thus, sexual mimicry may provide evidence that selection acting on reproductive success can favor coordinated evolution of neural and reproductive systems, supporting the hypothesis that shared genetic, developmental, and molecular mechanisms link cognitive and reproductive functions.

## 7. Sex Change and Sexual Selection

Sexual selection can drive profound evolutionary changes in reproductive strategies. In many reef and coastal fishes, mating success depends strongly on body size, social dominance, and access to mates. Sex change evolves when switching sex maximizes lifetime reproductive output, as predicted by the size-advantage model [27]. Hermaphroditism, defined as the expression of both male and female reproductive functions within a single individual, occurs in more than 450 fish species and may be either simultaneous or sequential [27]. Sequential hermaphroditism is particularly relevant to sexual selection because sex change is often triggered by social cues that alter mating opportunities. A well-studied example is the bluehead wrasse, *Thalassoma bifasciatum*, in which females rapidly transition to males following the loss of a dominant male [28]. This transition begins with immediate behavioral changes and the establishment of social dominance within one to two days, followed by endocrine shifts marked by declining estrogen levels and rising androgen concentrations. Ovarian regression occurs within several days, testicular tissue develops shortly thereafter, and functional spermatogenesis is achieved within approximately one week. Complete male coloration and reproductive competence develop within weeks, allowing newly transformed males to compete successfully for mates. This process highlights how sexual selection can shape not only courtship behaviors and secondary sexual traits, but also the sexual phenotype itself in response to social and competitive environments. Todd et al. reported that socially induced sex change in bluehead wrasse is triggered by stress-related pathways, notably the activation of the cortisol signaling axis, followed by the repression of aromatase and extensive epigenetic reprogramming [28]. Transcriptomic and methylome analyses indicate that ovarian tissue is not directly converted into testicular tissue, but instead passes through a transient, pluripotent-like intermediate state before testis development, highlighting the central role of hormonal and epigenetic mechanisms in mediating socially driven sex change [28].

Importantly, these socially induced changes are mediated by molecular mechanisms that include activation of stress response pathways, repression of aromatase, large-scale changes in gene expression, and extensive epigenetic reprogramming of the gonads. The discovery that ovarian tissue transitions through a pluripotent-like intermediate state before becoming testicular tissue demonstrates how environmental and social cues can directly influence reproductive phenotype through coordinated molecular processes. Thus, sex change provides evidence that social and behavioral environments can regulate reproductive function through hormonal, genetic, and epigenetic mechanisms, illustrating a possible link between behavior, neural processing of social signals, and reproductive biology. Overall, while sex change systems provide powerful models for sexual selection, stronger causal and cross-level mechanistic evidence is still needed to fully connect social environment, molecular regulation, and reproductive fitness.

## 8. Molecular Mechanisms Supporting the Sexual Selection Theory

Behavioral evidence for sexual selection provides an important framework for investigating the underlying molecular mechanisms, as sexually selected traits are ultimately mediated by genes and signaling pathways that regulate neural and reproductive functions. Behaviors such as mate choice, courtship, social dominance, and mating performance arise from neural circuits whose development and activity are controlled by specific genetic and molecular networks. If these networks also influence reproductive processes, then selection acting on behavioral traits may simultaneously affect reproductive function. Consequently, evidence that the brain and reproductive tissues share conserved genes, pathways, and regulatory mechanisms would provide a molecular basis linking sexual selection, cognition, and reproductive success.

Because sexual selection may suggest a possible evolutionary link between cognitive performance and reproductive success, the brain and sperm or testis are under coordinated selection to optimize behaviors and physiological traits that enhance mating success. Neural processes governing decision-making, social dominance, and courtship must be tightly integrated with testicular functions such as spermatogenesis, hormone production, and sperm performance, favoring shared cellular and molecular pathways, including epigenetic regulation, stress signaling, and energy metabolism. Therefore, we hypothesize that selection acting on cognition can indirectly affect reproductive tissues, and vice versa, thereby promoting conserved mechanisms that jointly support behavioral and fertilization success.

Table 1 summarizes studies highlighting shared functions and molecular mechanisms between the reproductive and nervous systems. A striking similarity between neurons and sperm cells is the presence of many neural receptors in sperm, which may contribute to sperm motility and the acrosomal reaction, an essential event for fertilization [29]. For example, the γ-Aminobutyric acid type A receptors (GABAARs) are the primary inhibitory neurotransmitter receptors in the central and peripheral nervous system and are major targets for various drugs. The GABAARs have been detected in the sperm membrane of several species, including humans, pigs, and sheep, and may play roles in sperm motility and fertilization [29]. Exocytosis is an essential process in neural function, in which membrane-bound vesicles fuse with the plasma membrane to release their neurotransmitter contents into the extracellular space. Several neurotransmitters, including adrenergic receptors, GABA_A_ receptor channels, serotonin receptors, and glycine receptors, are present in sperm and involved in the acrosomal reaction, an exocytotic event essential for fertilization [29]. Notably, both sperm and neurons are enriched in polyunsaturated fatty acids (PUFAs), which serve critical roles in cellular function. In the brain, PUFAs are essential for neurodevelopment, while in sperm, they are important for spermatogenesis and overall reproductive function [30].

Another neurotransmitter shared between neurons and sperm is catecholamines. In the central nervous system, catecholamines, such as dopamine and norepinephrine, serve as key neurotransmitters that regulate motor control, cognition, emotion, memory, and neuroendocrine processes. High concentrations of catecholamines have also been observed in the semen and oviduct of several species, indicating roles in sperm capacitation [31], motility [32], and the acrosomal reaction [33]. Urra et al. reported the presence of dopamine transported in stallion sperm and that high levels of dopamine reduce sperm motility and acrosomal integrity, suggesting a connection between cognition and the reproductive system [34]. Malorni et al. investigated the relationship between spermatogenesis and brain electrocortical activity in two mouse strains, finding that the C57 strain exhibited faster neural maturation than the SEC strain, which was associated with accelerated differentiation of the seminiferous epithelium [35].

Zinc provides a compelling example of how fundamental molecular requirements are shared between the nervous and reproductive systems. In both the brain and sperm, zinc contributes to signaling processes, structural organization, and protection against oxidative stress. Yet, these functions are expressed in tissue-specific contexts that support cognition and reproduction, respectively [36]. Proper zinc homeostasis is therefore critical, as excess zinc can induce neurotoxicity and reproductive toxicity, whereas deficiency compromises neural function and spermatogenesis [36]. Beyond its physiological roles, zinc also reveals more profound molecular parallels between these systems. Proteomic analyses of zinc-regulated proteins during sperm capacitation have uncovered enrichment of pathways classically associated with neurodegenerative disorders, including Huntington’s and Parkinson’s diseases [37]. These findings suggest that sperm and neural cells share some conserved genetic and molecular networks, consistent with the possibility that certain mechanisms involved in brain function and reproductive processes may be evolutionarily related and functionally interconnected. The involvement of sperm-active genes in brain disorders such as Huntington’s disease is not surprising, given that the brain and testis exhibit the highest expression of the disease gene, *HTT*, among all body tissues [38]. Furthermore, the *HTT* gene has known functions in neurogenesis, early embryonic development, ciliogenesis, vesicle trafficking, and transcriptional regulation [36]. Inactivation of the *Htt* gene in the forebrain and testis led to neuronal degeneration and sterility in mice [39].

Indeed, accumulating evidence suggests that mutations or abnormal expression of the same genes in both the brain and the testis across species (e.g., humans, mice, and cattle) can simultaneously cause dysfunction in these tissues [40,41,42]. Kitamura et al. showed that a mutation in the aristaless-related homeobox (*ARX*) gene, which is highly expressed in the brain and testis, caused abnormal development of the forebrain and testis in humans [40]. Serendipitously, we recently discovered in sheep that 24 of 35 sperm-active genes (e.g., *LPAR1*, *THOC1*, and *CTNNA3*) epigenetically altered by paternal diet are involved in brain development [5,6]. For example, the lysophosphatidic acid receptor 1 (*LPAR1*) gene was highly methylated in the sperm of methionine-supplemented rams and their subsequent F1 and F2 generations compared with controls [5]. *LPAR1* signaling can inhibit programmed cell death and enhance neurogenesis through premature cell cycle exit and neuronal differentiation [43]. *LPAR1* deletion is associated with neurodevelopmental disorders in humans and mice [42]. Furthermore, knockout of the *Lpar1* gene in mice resulted in azoospermia and age-related progression of germ cell degeneration [44], and a splicing mutation in *LPAR1* was significantly associated with semen quality in bulls [45].

The THO complex subunit 1 (*THOC1*) is a core component of the TREX (transcription/export) complex, which plays a central role in mRNA processing and nuclear export. *THOC1* has been implicated in both neural and reproductive functions. Maeder et al. demonstrated that *THOC1* is required for dopamine signaling and neuronal survival and is highly conserved from yeast to mammals [46]. Notably, genetic variation in *THOC1* has been associated with canine behavioral traits, including herding, predation, and temperament, highlighting its relevance to cognition and behavior [47]. In the reproductive system, Wang et al. showed that *Thoc1* deficiency in mice results in abnormal testis development. Another gene identified in our study that links sperm and brain function is *CTNNA3* [5,48]. Altered DNA methylation of *CTNNA3* has been associated with male infertility in humans [41], while genetic and epigenetic variation in this gene has also been linked to neurodevelopmental and neurodegenerative disorders, including autism and Alzheimer’s disease [49,50]. Together, these findings suggest that shared genes and pathways between the brain and the sperm may contribute to both neural function and reproductive disorders, reinforcing the concept of molecular integration between the brain and reproductive system.

Collectively, these observations have motivated efforts to examine the extent of shared gene and protein expression between the brain and reproductive tissues, including the testis and sperm. Identifying such shared molecular features is important because it reveals conserved mechanisms that jointly support cognition and reproduction, two traits tightly linked to fitness and sexual selection. Overlapping pathways provide a framework for understanding how selection acting on neural traits, such as learning, behavior, and social dominance, may simultaneously influence reproductive capacity, and vice versa. From a biomedical perspective, this molecular convergence may help explain the frequent co-occurrence of neurological disorders and infertility, as disruptions in shared genes or pathways can impair both systems. Moreover, because sperm are highly specialized yet experimentally accessible cells, their molecular similarity to neurons offers valuable opportunities for modeling complex neural processes, disease mechanisms, and the evolutionary integration of brain and reproductive functions [36]. However, this approach should be interpreted cautiously, as sperm and neurons differ substantially in cellular architecture, developmental origin, physiological function, and environmental context. Consequently, molecular similarities between these cell types are unlikely to capture the full complexity of neural networks, brain-specific regulatory mechanisms, or higher-order cognitive processes, limiting the extent to which findings from sperm can be generalized to the nervous system.

The evolutionary coupling of cognition and reproductive performance would necessitate high levels of gene expression sharing between neural and reproductive tissues. Indeed, transcriptomic analyses across multiple human and mouse tissues have shown that the brain and testis share more similar gene expression profiles than any other tissue pair [51], and large-scale proteomic studies have revealed that these two organs also exhibit the greatest similarity in protein expression among diverse tissues [52]. Another compelling molecular example linking cognition and reproductive performance is the extensive chromatin remodeling that occurs during spermatogenesis, where most histones are replaced by protamines. Although this transition enables the extreme compaction required for sperm function, approximately 1–15% of nucleosomes are retained at specific genomic regions, including developmental gene promoters and imprinted loci. These retained nucleosomes are enriched for regulatory histone modifications such as H3K4me3, H3K27me3, and H3K9me3, creating epigenetic domains that can persist after fertilization and influence early embryonic gene expression [53]. Importantly, many of the genes associated with retained sperm nucleosomes are involved in neural development, neurogenesis, and transcriptional regulation of the central nervous system. Neurodevelopment is particularly sensitive to epigenetic perturbations during early embryogenesis, a period that partially overlaps with primordial germ cell specification and migration [54]. Moreover, the central nervous system is among the first organ systems to be specified and provides a developmental framework that guides subsequent organogenesis [54]. The shared dependence of both germ cells and neural tissues on precise chromatin remodeling, epigenetic programming, and transcriptional regulation highlights a mechanistic link between reproductive success and cognitive development. From an evolutionary perspective, the conservation of these epigenetic pathways suggests that selection for reproductive fitness may have simultaneously influenced molecular systems that support neural development and cognitive function, providing a tangible connection between reproductive biology and the evolution of complex behavioral traits. 

Despite these advances, the evolutionary conservation of the brain–testis transcriptional relationship across species, as well as its relationship to the ovary, has remained largely unexplored. To address these gaps, we examined shared gene and protein expression across the brain, testis, sperm, and ovary using a combination of experimental and in silico approaches. Proteomic and transcriptomic data were generated experimentally in *Ovis aries* (sheep) and *Rattus norvegicus* (rat) and complemented with transcriptomic analyses from *Mus musculus* (mouse), *Macaca mulatta* (rhesus macaque), and *Homo sapiens*. We identified 8464 protein-coding genes shared between the brain and sperm or testis, the majority of which showed conserved expression patterns across all five species. In rats, most of these genes were also shared with the ovary, indicating a broader reproductive–neural network. Functional annotation revealed that many shared genes are involved in both neurological and reproductive processes, highlighting a deeply conserved genetic architecture linking cognition and reproduction [7].

**Table 1:** Molecular mechanisms involved in neurological and reproductive functions.

Reference	Molecular Mechanisms Shared Between Sperm and Neurons	Species
Meizel (2004) [29]	γ-Aminobutyric acid type A receptors (GABAARs), major inhibitory neurotransmitter receptors and drug targets, have been identified in the membranes of human, pig, and sheep sperm. These receptors are thought to play roles in sperm motility and fertilization.	Human, pig, sheep
Pierce et al. (2009) [30]	Both sperm and neurons are enriched in polyunsaturated fatty acids (PUFAs), which are essential for neurodevelopment in the brain and for spermatogenesis and reproductive function in sperm.	Mammals
Adeoya-Osiguwa et al. (2006) [31]	Alpha2- and beta-adrenergic receptors, components of the autonomic nervous system, are expressed in mammalian spermatozoa and play roles in inhibiting spontaneous acrosome reactions and maintaining fertilizing ability.	Mammals
Bavister et al. (1979) [32]	Catecholamines, including dopamine, norepinephrine, and epinephrine, are essential neurotransmitters and hormones that act as cofactors for sperm motility and are required for hamster sperm motility in vitro.	Rodents
Way and Killian (2002) [33]	The presence of norepinephrine, a crucial neurotransmitter and stress hormone, in the bovine oviduct suggests catecholamines contribute to fertilization-related events. Norepinephrine also regulates capacitation and induces the acrosome reaction in bull spermatozoa.	Cattle
Urra et al. (2014) [34]	Dopamine, involved in functions related to cognition, emotions, and control of motor activity, can regulate sperm function, specifically acrosomal integrity and sperm motility.	Equine
Lawlor et al. (2022) [36]	Zinc supports signaling, structural integrity, and antioxidant defense in both neural and reproductive systems. Maintaining zinc homeostasis is essential, as an imbalance impairs cognition and spermatogenesis, while excess zinc can cause neurotoxicity and reproductive toxicity.	Human, mice
Sharp and Ross (1996) [38]; Dragatsis et al. (2000) [39]	The Huntington disease gene, *HTT*, is highly expressed in the brain and testis and functions in neurogenesis, embryonic development, ciliogenesis, and transcriptional regulation. Htt inactivation in the mouse forebrain and testis causes neuronal degeneration and sterility.	Human, mice
Kitamura et al. (2002) [40]	Aristaless-related homeobox (*ARX*) gene is essential for neuronal proliferation, migration, and differentiation during forebrain development and also contributes to testis differentiation. *ARX* mutations cause neurological disorders, including epilepsy, intellectual disability, and X-linked lissencephaly.	Human
Gross et al. (2020) [6]; Braz et al. (2022) [5]	Methionine supplementation in rams from weaning to puberty altered thousands of sperm DNA methylation sites, with most affected genes involved in brain development and neural functions.	Sheep
Kamiński et al. (2018) [45]	The lysophosphatidic acid receptor 1 (*LPAR1*) gene regulates neurogenesis and cell survival, while its disruption is associated with neurodevelopmental disorders, azoospermia, germ cell degeneration, and reduced semen quality.	Mice, cattle
Man et al. (2017) [55]; Slegtenhorst-Eegdeman et al. (1998) [56]	*FMR1*, the gene underlying fragile X syndrome and inherited intellectual disability, is also linked to ovarian dysfunction in women and regulates Sertoli cell proliferation during testicular development in mice.	Mice, Human

## 9. Concluding Remarks

In summary, the reviewed information supports the idea that Darwin’s theory of sexual selection, which explains the evolution of traits that enhance reproductive success through female mate choice or male–male competition for mates, acts on integrated molecular networks that jointly shape cognitive performance and reproductive success. We also demonstrate that the brain, sperm, testis, and ovary share a substantial number of genes and molecular pathways, many of which are highly conserved across species. These results suggest a shared molecular architecture underlying reproductive and neural processes could be affected by sexual selection. However, it is important to note that much of the current evidence supporting a neural–reproductive connection remains indirect. Future research should prioritize experimental validation of the functional roles of shared genes through gene knockout and gene-editing studies, elucidate their regulatory and epigenetic mechanisms, and determine how selection on cognitive traits feeds back onto reproductive fitness in mammals across evolutionary timescales. In addition, analyses of selection signatures and comparative studies across diverse species will be essential for assessing whether shared genetic and molecular mechanisms have been evolutionarily conserved and whether they causally contribute to both neural and reproductive phenotypes.

## Data Availability

No new data were created or analyzed in this study. Data sharing is not applicable to this article.
